# The morbidity and prevalence of mental pathology in children and adolescents in the kyrgyz republic for 2005-2020

**DOI:** 10.1192/j.eurpsy.2024.535

**Published:** 2024-08-27

**Authors:** A. Nurali Kyzy, T. M. Kadyrova

**Affiliations:** ^1^medical psychology, psychiatry and addiction, I.K. Ahunbaev Kyrgyz State Medical Academy; ^2^childrens department, Republican Center for Psychiatry and Narcology; ^3^member, Kyrgyz Psychiatric Association, Bishkek, Kyrgyzstan

## Abstract

**Introduction:**

One of the principles of healthcare is preventive focus, that is, the implementation of measures to improve the hygienic education of the population and maintain a healthy lifestyle. The scientific rationale for carrying out primary prevention activities is based on an analysis of morbidity and prevalence rates and their dynamics.

**Objectives:**

to conduct a comparative analysis of the primary incidence and prevalence of mental pathology in children and adolescents (0-17 years) in the Kyrgyz Republic for 2005-2020 .

**Methods:**

statistical data from the Republican Center for Electronic Health and the National Statistical Committee of the Kyrgyz Republic were used  (http://www.stat.kg/ru/rss/), (http://cez.med.kg/).

**Results:**

primary incidence of mental pathology among children and adolescents in 2005, 2010, 2015 and 2020 amounted to 66.2, 44.1, 44.8, 51.1 respectively (based on 100,000 the child population). The prevalence of mental pathology for 2005-2020 was 418.4, 317.0, 312.5, 400.0 respectively (based on 100,000 the child population). That is, morbidity and prevalence rates show higher numbers in 2005 and in 2020 (Diagram №1). In the gender aspect, morbidity rates were higher in males 40.0, 28.0, 31.3, 31.0 compared to females 26.3, 16.1, 17.1, 20.1 (based on 100,000 the child population; (Diagram №2).

**Image:**

**Figure:**
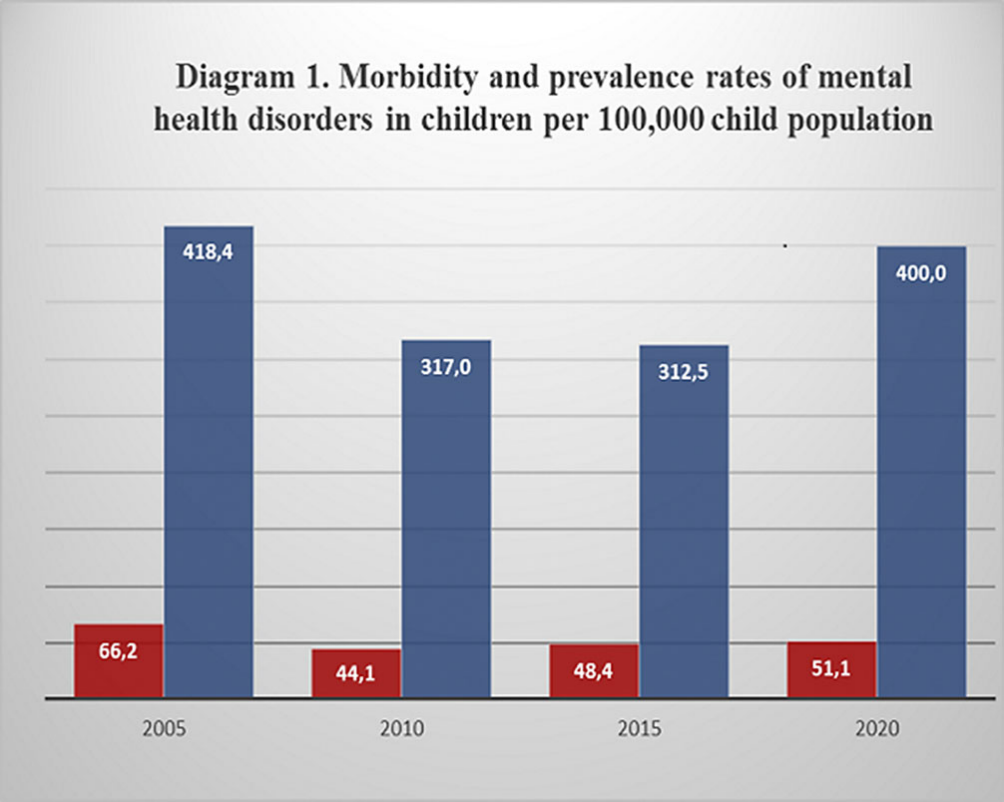

**Image 2:**

**Figure:**
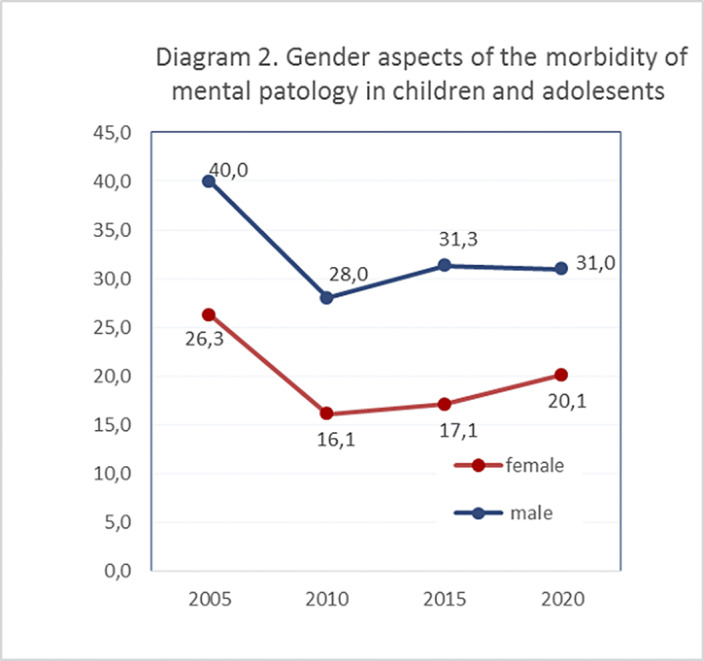

**Conclusions:**

the variability of the obtained indicators of morbidity and prevalence of mental pathology in children and adolescents is due to difficulties in providing specialized psychiatric care to the child population due to the lack of child psychiatrists in the regions of the country, the processes of population migration, and the phenomenon of stigmatization. In this regard, measures and educational programs are needed to improve the provision of psychiatric care to the child population at the level of primary medical and social care.

**Disclosure of Interest:**

None Declared

